# Silent for Ten Years: Late Flexor Pollicis Longus Rupture Following Volar Plate Fixation of the Distal Radius

**DOI:** 10.7759/cureus.111055

**Published:** 2026-06-17

**Authors:** Dylan Wood, Marco Posada Rodriguez, Lloyd Blythe, Millan Shah, Sarthak Chakravarty, Gary Schwartz

**Affiliations:** 1 Medical Education, Nova Southeastern University Dr. Kiran C. Patel College of Allopathic Medicine, Fort Lauderdale, USA; 2 Medicine, Nova Southeastern University Dr. Kiran C. Patel College of Osteopathic Medicine, Fort Lauderdale, USA; 3 Orthopaedic Surgery, Nova Southeastern University Dr. Kiran C. Patel College of Allopathic Medicine, Fort Lauderdale, USA

**Keywords:** flexor digitorum superficialis tendon transfer, flexor pollicis longus (fpl), flexor tendon rupture, fracture of distal radius, orif distal radius

## Abstract

Flexor pollicis longus (FPL) tendon rupture is an uncommon complication following volar locking plate fixation for distal radius fractures and is most often attributed to attritional wear from hardware contact. While most reported cases occur within the first few years after open reduction and internal fixation (ORIF), rupture occurring a decade or more after surgery remains exceedingly rare. A 48-year-old, left-hand-dominant male field engineer presented with a two-week history of inability to flex the interphalangeal (IP) joint of his left thumb. The symptoms began acutely while lifting heavy boxes at work and were preceded by sharp wrist pain. Ten years prior, he underwent ORIF of a left distal radius and ulnar styloid fracture after he fell from a ladder. He had remained asymptomatic throughout the years following surgery. Physical examination demonstrated complete loss of active thumb IP flexion. A flexor digitorum superficialis tendon to the ring finger (FDS-4) to the FPL tendon transfer was performed. The postoperative functional outcome after such a tendon transfer depends on factors such as surgical technique, tendon selection, and patient compliance with therapy, but it can often result in meaningful restoration of function.

This case emphasizes the importance of long-term patient counseling regarding warning signs of hardware-related attritional tendon rupture, such as new-onset volar wrist pain, clicking, or sudden loss of thumb IP flexion, even years after fracture fixation. It also demonstrates that attritional FPL rupture following volar plate fixation may occur up to a decade postoperatively, requiring long-term patient counseling and continued clinical awareness for delayed tendon complications.

## Introduction

The flexor pollicis longus (FPL) is the only extrinsic flexor of the thumb interphalangeal (IP) joint. FPL ruptures occur most commonly due to laceration injuries, causing a partial or complete disruption of the tendon [1]. However, due to the FPL’s course running closely over the volar aspect of the distal radius and implanted hardware, attrition injuries are a rare but well-defined complication arising from open reduction and internal fixation (ORIF) of the distal radius. A patient who sustains FPL rupture can present with a loss of active flexion of the thumb IP joint [2].

The FPL is one of the three muscles found in the deep anterior compartment of the forearm. It originates at the anterior radius and at the radial half of the interosseous membrane, forming a large, singular tendon that passes through the carpal tunnel to insert at the volar surface of the base of the distal phalanx of the thumb [3]. When primary FPL repair cannot be performed, three options may be considered as an alternative: free tendon graft, flexor digitorum superficialis (FDS) tendon transfer, and thumb IP joint arthrodesis. Free tendon grafts are considered when there is a favorable tendon bed and there is adequate muscle excursion. The advantages are that this procedure preserves the IP joint motion without causing donor finger morbidity, and its disadvantages are requiring a healthy tendon bed, risking tendon adhesion, and occasionally requiring a two-stage procedure. FDS tendon transfer is considered when there is a favorable tendon bed, but there is a mobility issue with the native FPL. Its advantages are that it may restore thumb flexion strength when the native FPL is unable to contract sufficiently, and its disadvantages involve sacrificing a donor finger’s FDS. Zukawa et al. proposed intraoperative assessment of the flexor tendon's passive and active motion to guide decision-making between a free tendon graft or tendon transfer [4,5]. Thumb IP joint arthrodesis is considered a salvage procedure in cases such as severe tendon-bed scarring, failed reconstruction, or arthritis. Sacrificing thumb mobility in exchange for stability provides advantages such as predictability, and its disadvantages are permanent loss of thumb IP motion and thumb flexion [6,7].

Prior literature has established attritional FPL rupture as a recognized complication of volar plate fixation, often due to hardware prominence or tendon contact with the distal plate edge [2]. A useful radiographic classification tool was presented by Soong et al. in 2011 to assess the prominence of a volar locking plate relative to the watershed line of the distal radius [8]. This proposed grading system has been shown to be a strong predictor of tendon irritation/rupture [9,10]. However, reports of rupture presenting a decade or more after fixation remain limited [11]. This paper highlights the potential for volar plate fixation to carry a long-term risk of attritional FPL rupture. This case report offers a focused synthesis of other case reports and will serve as evidence for the long-term risk of FPL ruptures following ORIF of the distal radius. Surgeons should take this long-term complication into account when deciding surgical management. This report compares the present case with findings reported in prior studies on late tendon attrition after distal radius fixation and reinforces the need to recognize FPL rupture as a complication that may emerge long after apparent fracture healing.

## Case presentation

The patient is a 48-year-old left-hand-dominant man who worked as a field engineer. He presented to the emergency department with pain in the left wrist for two weeks associated with some tingling in the thumb, index, and long fingers lasting four days. The injury occurred when the patient fell from a ladder and the ladder landed on his wrist. He has not had any previous similar problems. The patient was a non-smoker and did not drink any alcohol or use any illicit drugs. No other medical comorbidities were present. On physical examination, there was swelling and tenderness along the dorsal and volar aspects of the left wrist and hand, as well as decreased digital motion. Two-point discrimination in all the digits was 6 mm. There was no evidence of thenar atrophy. Thenar sensation was normal. There was a negative percussion test at the carpal tunnel. X-rays demonstrated a comminuted intra-articular, displaced fracture of the left distal radius in association with a fracture of the base of the ulnar styloid. There was no disruption of Gilula’s arcs on the anterior-posterior view, which indicated that there was no evidence of carpal instability. There was no loss of collinearity between the lunate and capitate on the lateral view (Figure 1). A closed reduction was performed, and the patient was placed into a long-arm splint. The tingling in the digits resolved after the reduction was completed. He was then referred to an orthopedic hand surgeon.

**Figure 1:**
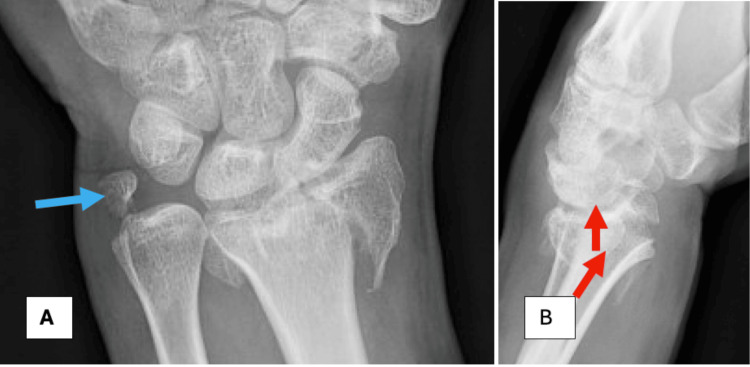
Pre-operative radiographs of the left wrist. Anterior posterior (A) and lateral (B) radiographs of the left wrist demonstrate an impacted, comminuted fracture of the distal radius. Note the dorsal tilt of the distal articular surface of the radius (red arrows) on the lateral view. An associated displaced fracture of the ulnar styloid is present (blue arrow).

A CT scan of the left wrist demonstrated the displaced, intra-articular fracture of the distal radius and the ulnar styloid (Figure 2).

**Figure 2:**
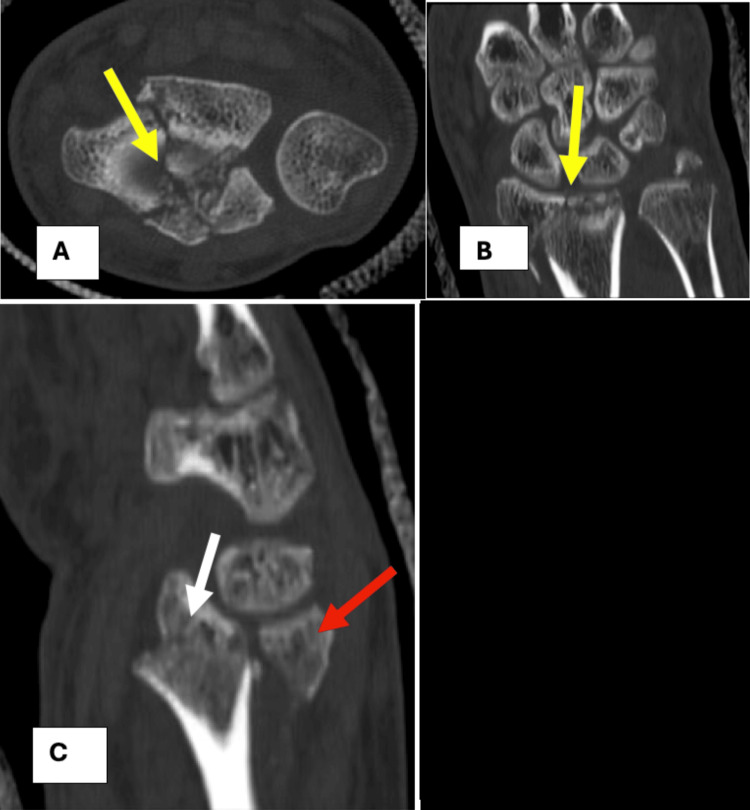
Pre-operative CT scan of the left wrist. Axial (A), coronal (B), and sagittal (C) non-contrast CT images of the wrist confirm the comminuted nature of the radius fracture. Note the extension of a fracture line to the articular surface (yellow arrows), the large, displaced fracture fragment at the dorsal aspect of the radius (red arrow), and the dorsal tilt of the articular surface of the radius (white arrow).

The patient subsequently underwent ORIF of the left distal radius and ulnar styloid fractures on day 4 after the injury (Figure 3).

**Figure 3:**
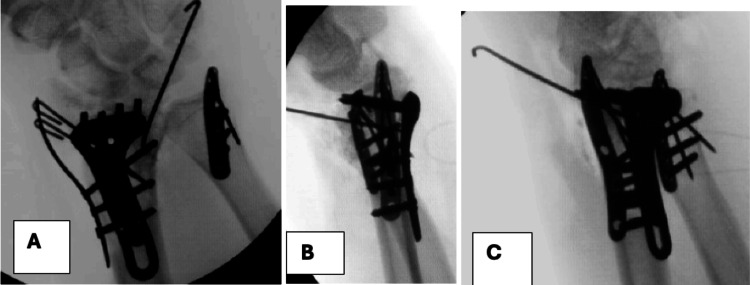
Post-operative radiographs of the left wrist. Post-operative fluoroscopic anterior posterior (A), lateral (B), and oblique (C) images show fixation of the distal radius and ulnar styloid fractures.

The ulnar styloid was fixed due to some laxity in the distal radio-ulnar joint after fixation of the distal radius fracture. A volar radial incision was utilized to approach the distal radius fracture. A dorsal ulnar incision was utilized to approach the ulnar styloid fracture. The distal radius fracture was transfixed using a volar-bearing and a radial column plate. The plate was located at the level of the critical line and proximal to the volar rim, consistent with a Soong index of one. The ulnar styloid was fixed using a fragment-specific plate. The patient was placed into a dorsal and volar short-arm splint after the surgery. Occupational hand therapy was started on post-op day 2. This consisted of active, active-assisted, and passive range of motion; retrograde massage; edema reduction techniques; activities of daily living; therapeutic activities; and a home-therapy program. The home program consisted of three phases, which are described in Table 1.

**Table 1:** The home therapy program prescribed to the patient.

Weeks	Activity
1-2	Active and passive range of motion to the digits, wrist, and forearm; edema control; and retrograde massage
3-6	Therapeutic activities such as buttoning, tying shoes, zipping, use of clothes pine, and therapeutic putty
>6	Strengthening exercises

The patient was discharged from occupational therapy at three months post-op. He continued his home exercise program until five months post-op, at which time he was discharged from active orthopaedic care. At the time of discharge, active range of motion of the left wrist was 0-45 degrees of dorsiflexion, 0-45 degrees of volar flexion, 0-20 degrees of radial deviation, and 0-30 degrees of ulnar deviation. His grip strength on the Jamar dynamometer was 45 pounds compared to 85 pounds on the contralateral right hand. All fingertips reached the distal palmar crease. The patient’s timeline from injury, surgery, and recovery is seen in Table 2.

**Table 2:** Timeline of the clinical course.

Days	Event
0	Injury date
4	Surgery date
6	Occupational hand therapy began
18	Wrist-controlled splint applied
39	Wrist-controlled splint discontinued
96	Occupational therapy discontinued
154	Home therapy program discontinued and patient discharged from active care

The patient presented to the operative surgeon 10 years post op with a chief complaint of pain in the left wrist and inability to flex the IP joint of the left thumb for a two-week period. He noticed this when he was at work moving some heavy boxes. He first noticed pain in the wrist and then an inability to flex the IP joint of the left thumb (Figure 4).

**Figure 4:**
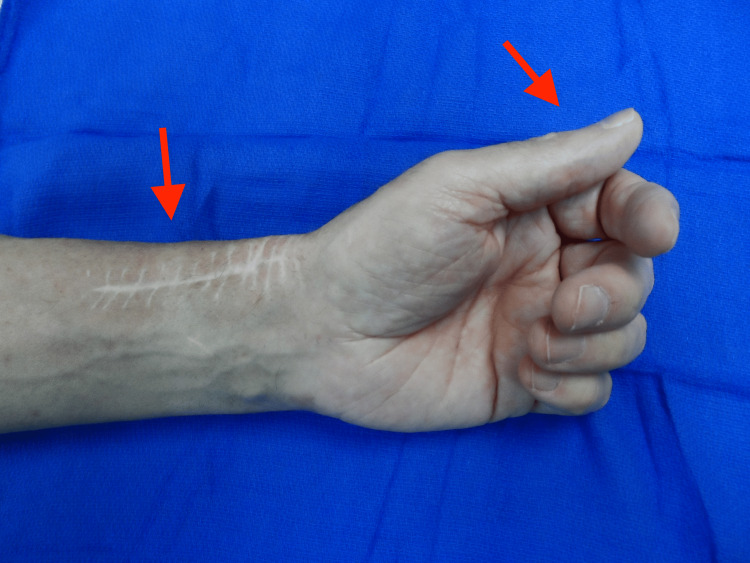
Pre-operative photo of the wrist and hand. Pre-operative photograph demonstrating the healed incision from the previous surgery and the inability to actively flex the left thumb (red arrows).

He had not had any problems with the wrist since he was discharged from active care 10 years previously. Radiographs did not demonstrate any loosening of the hardware. An MRI demonstrated a rupture of the FPU tendon with retraction (Figure 5).

**Figure 5:**
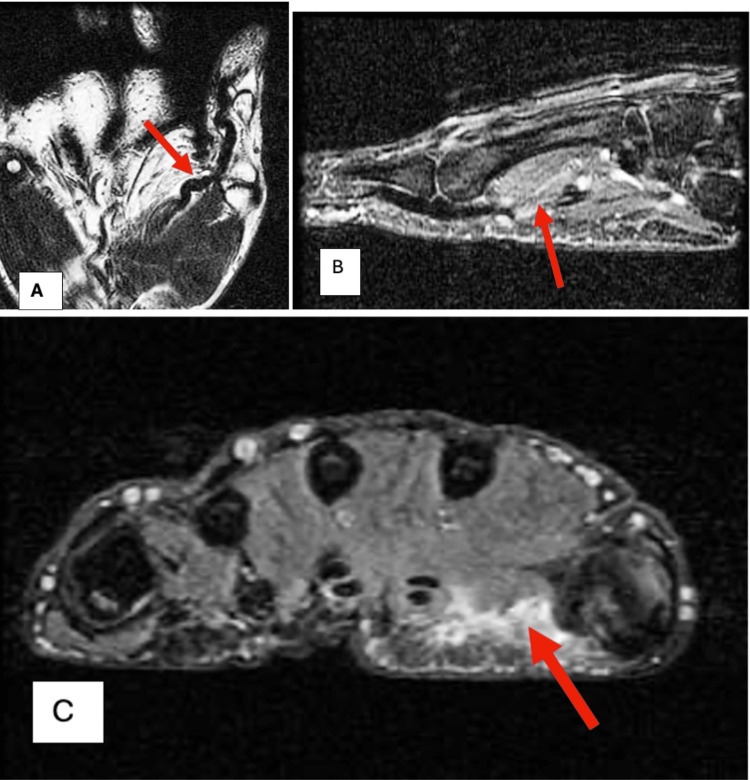
Pre-operative MRI of the left wrist and hand. Pre-operative coronal (A), sagittal (B), and axial (C) MRI images of the left hand and wrist demonstrate the rupture of the flexor pollicis longus (FPL) tendon with retraction (red arrows).

The patient was taken to surgery, at which time, the hardware was removed from the distal radius except for a broken screw (Figure 6).

**Figure 6:**
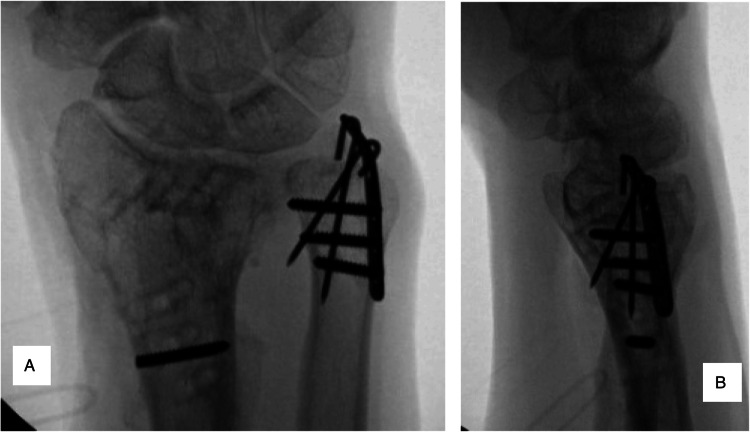
Intra-operative radiographs of the left wrist. Fluoroscopic anterior-posterior (A) and lateral (B) images demonstrate the distal radius hardware removed except for a broken screw, with maintenance of the distal ulnar hardware.

The FPU was initially explored in the distal forearm and was noticed to be totally fibrosed. The FDS of the left ring finger was transferred to the left FPU tendon, along with a carpal tunnel release. This was performed to make sure that the placement of the transfer was dorsal to the median nerve and as a vector-changing incision. This procedure was performed under general anesthesia. A tourniquet was used during the dissection portion of the procedure, which was 110 minutes. The surgical incisions are noted in Figure 7.

**Figure 7:**
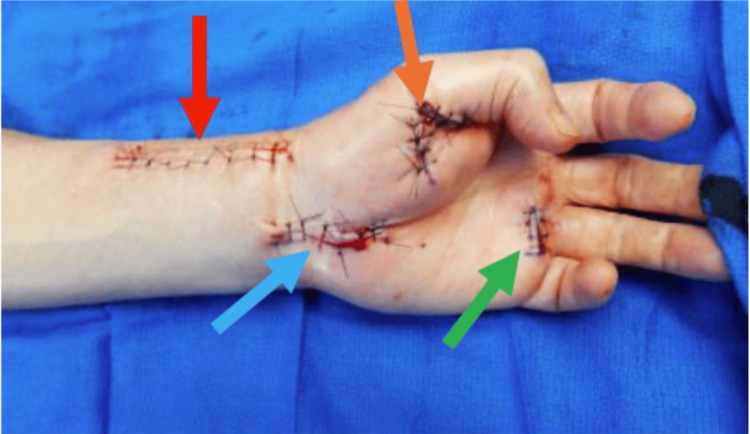
Incisions used for the tendon transfer. The volar radial wrist incision was used to explore the FPL tendon in the distal forearm (red arrow). The volar wrist incision was used to identify the median nerve and used as a vector-changing incision (blue arrow). The transverse incision along the base of the ring finger was used to identify the flexor digitorum superficialis (FDS) tendon (green arrow). The incision along volar aspect of the thumb was used to suture the transposed FDS to the FPL tendon (orange arrow).

The patient was placed in a short-arm volar and radial gutter splint, maintaining the wrist in 30 degrees of volar flexion, with the thumb in palmar abduction and the metacarpophalangeal and IP joints of the thumb in 30 degrees of flexion. The patient was seen by the occupational hand therapist at one week post-op, at which time he was placed into a custom-fabricated low-temperature thermoplastic flexor assist splint with an extension block (Figure 8).​​

**Figure 8:**
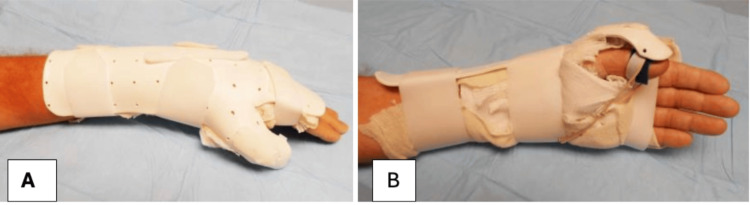
The post-operative splint fabricated by the occupational hand therapist. Post-operative splint showing the dorsal (A) and volar (B) aspects of the thermoplastic splint keeping the wrist, metacarpophalangeal, and interphalangeal (IP) joints of the thumb at 30 degrees of flexion.

Passive range of motion to all the thumb joints was begun. The IP joint of the thumb was manipulated through a full range of motion with the metacarpophalangeal joint blocked. At home, the patient began active extension and passive flexion of the IP joint of the thumb. At four weeks post-op, the splint was modified to place the wrist at 0 degrees of flexion. Strengthening exercises were started at six weeks post-op. At eight weeks post-op, the splint was discontinued. The patient continued with the occupational hand therapy for three months, at which time he was discharged from active care. At the time of discharge, his active range of motion of the left wrist was 0-45 degrees of dorsiflexion, 0-45 degrees of volar flexion, 0-20 degrees of radial deviation, and 0-30 degrees of ulnar deviation. The final active motion of the IP joint of the thumb at the time of discharge was 0-60 degrees of flexion (Figure 9).​​​​​​​

**Figure 9:**
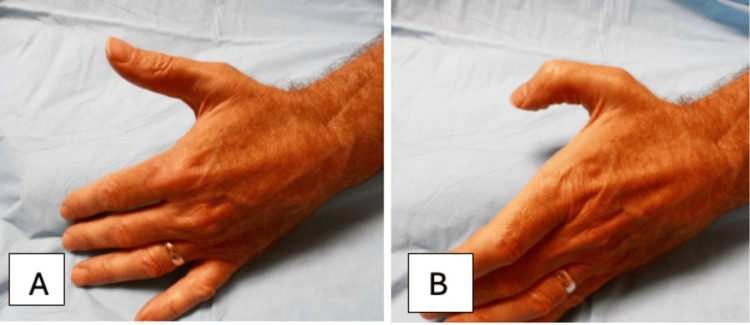
The final active range of motion of the thumb. The final active motion of the IP joint of the thumb is full extension (A) to 60 degrees of flexion (B).

His grip strength on the Jamar dynamometer was 50 pounds compared to 80 pounds on the contralateral right hand. The active digital range of motion was to the distal palmar crease. The patient’s timeline from injury, surgery, and recovery is seen in Table 3.​​​​​​​

**Table 3:** Timeline of the clinical course. IP: interphalangeal.

Day	Event
0	Day of surgery
7	Thermoplastic splint applied to wrist, metacarpophalangeal, and IP joints in 30 degrees of flexion, with the thumb in palmar abduction. Occupational hand therapy began
28	Splint modified to keep wrist and digits at 0 degrees flexion
42	Strengthening exercise program began
56	Splint discontinued
84	Occupational therapy discontinued. The patient was discharged from active care

## Discussion

Among upper extremity injuries, distal radius fractures are the most common fracture encountered. Volar locking plate fixation is a widely used treatment for these fractures, and the frequency of the use of these plates has more than doubled over the past decade. Tendon rupture rates postsurgically have been reported as high as 12%. The most commonly ruptured tendon after volar plate fixation is the FPL [12-15].

The mechanism behind rupture of the FPL is believed to involve chronic attritional wear from repetitive tendon contact with hardware along the volar aspect of the distal radius. This can be accentuated by plate prominence, screw prominence, increased tendon contact pressure, and repetitive tendon gliding. Tanaka et al. [16] investigated the contact pressure between the volar plate and the FPL tendon on seven cadaveric upper extremities relative to the watershed line or the most distal, transverse bony ridge on the volar surface of the radius. This experiment supported the theory that plates placed distal to the watershed line have an increased risk of impinging on the FPL tendon. Similar results were found in a study by Zelenski et al. [17], which concluded that plates placed distal to the watershed line have an increased contact pressure and tendon wear in cadaveric models. Volar plates exceeding 2 mm in thickness and placed less than 3 mm proximal to the watershed line are associated with a high risk of direct FPL tendon contact [18].

Most FPL ruptures occur within the first few years postoperatively [19]. Delayed presentations beyond this timeframe are rare, making the present case particularly unusual due to the 10-year interval from ORIF to FPL rupture. Previous cases have discussed the association between delayed FPL rupture and prominent volar plates and retained hardware [20,21]. Nunley and Rowan discuss a case of delayed rupture of the tendon with volar plate placement distal to the watershed line, highlighting the importance of plate positioning to prevent tendon irritation [20]. Similarly, Klug et al. described rupture of the FPL tendon after volar-fixed-angle plating due to tendon attrition against hardware [21]. Delayed tendon rupture caused by chronic irritation has also been described in non-hardware-related conditions such as scaphoid non-union and foreign bodies [22,23]. The present case supports the concept that chronic attritional degradation of tendons can remain clinically silent for many years before culminating in a tendon rupture, even in asymptomatic patients without radiographic evidence of hardware loosening.

FPL rupture typically presents with a sudden inability to flex the interphalangeal joint of the thumb, often accompanied by volar wrist pain [24]. In the present case, the patient was lifting heavy boxes when he felt an acute onset of wrist pain, followed shortly after by loss of thumb interphalangeal flexion. He had remained asymptomatic for 10 years following the fixation procedure and denied any significant interval trauma. The physical exam demonstrated loss of active thumb flexion, raising suspicion for an FPL tendon rupture. Radiographs displayed maintained alignment of hardware with no evidence of hardware loosening or failure. MRI confirmed FPL rupture with proximal tendon retraction. Advanced imaging modalities such as MRI and ultrasound have been shown to be useful in evaluating the integrity of tendons and may be useful in identifying attritional changes prior to tendon rupture [25]. The delayed presentation of this case emphasizes the importance of maintaining suspicion for tendon rupture in patients with prior distal radius ORIF presenting with acute thumb dysfunction, even years after initial surgery.

Management of FPL rupture is dependent on the condition of the tendon and the chronicity of the injury at the time of surgery [12]. Primary repair may be feasible in acute ruptures with minimal retraction; however, chronic attritional ruptures are associated with degeneration, fibrosis, and retraction that limits the ability for direct repair. In the present case, intraoperative exploration displayed an irreparable FPL tendon with severe fibrosis within the distal forearm. Subsequently, reconstruction was performed using the FDS as a tendon transfer to the FPL tendon. This transfer has been well described as a reliable option for restoration of thumb flexion [26]. A carpal tunnel release was also performed to allow safe tendon passage dorsal to the median nerve. Following postoperative immobilization and occupational hand therapy, the patient achieved restoration in active interphalangeal thumb flexion. These findings are consistent with previously reported cases of FDS to FPL tendon transfer [26].

This case highlights the importance of several clinical considerations for surgeons performing volar plate fixation for distal radius fractures. The potential for severe functional repairment from FPL tendon rupture warrants careful attention to plate positioning and hardware prominence during the index procedure [14,19]. The present case demonstrates that FPL tendon rupture can occur several years after surgery. This prolonged interval suggests that chronic low-grade tendon irritation may progress to complete tendon rupture over time. Surgeons should therefore maintain long-term awareness of this complication and counsel patients to seek evaluation if they develop volar wrist pain, thumb weakness, or loss of flexion of the thumb. In select patients with high-risk plate positioning, elective hardware removal may reduce the risk of tendon complications [19,27]. Early recognition and timely intervention remain essential for optimal functional outcomes.

There are several limitations to this case report. First, the findings are inherently limited in generalizability due to it being a single case report. Due to this, causation between retained hardware and delayed FLP rupture cannot be established. Additionally, no interval ultrasound or MRI surveillance was done during the 10-year postoperative period, making it not possible to determine the onset or progression of tendon degeneration prior to FPL rupture. Although the patient in this report achieved satisfactory functional recovery following FDS-to-FPL tendon transfer, long-term postoperative outcomes beyond discharge were unavailable. Furthermore, patient-reported outcome measures were not obtained. Additionally, the retrospective nature of this case report has the potential for incomplete documentation. Despite the limitations of this study, the unusual, delayed presentation of the FPL rupture and successful surgical reconstruction contribute valuable information to the limited literature regarding late attritional flexor tendon rupture following distal radius volar plating.

## Conclusions

This unique case demonstrates that FPL tendon rupture secondary to attritional wear from volar plate fixation of distal radius fractures can occur as late as 10 years postoperatively. In the absence of symptoms or wear related to the volar plate fixation, this case underscores the importance of educating patients about warning signs of tendon irritation. This includes new-onset volar wrist pain, clicking, or loss of thumb interphalangeal joint flexion, regardless of time elapsed. The current case further supports that using an FDS-4 tendon transfer is a reliable reconstructive option for chronic FPL rupture when the native tendon is no longer viable for primary repair or grafting. While our patient was asymptomatic for ten years, it was important to continue to observe the patient due to potential future problems. Although evidence is limited in case reports, it may be reasonable to consider discussing the potential risks of long-term hardware retention with patients at the time of the initial fixation.

## References

[1] Pearce O, Brown MT, Fraser K, Lancerotto L (2021). Flexor tendon injuries: repair &amp; Rehabilitation. Injury.

[2] Lv YX, Chen MM, Su CX, Ye HN, Yang J, Li J (2021). Analysis of risk factors associated with flexor pollicis longus injury after volar plating of distal radius fractures. J Int Med Res.

[3] Standring S (2008). Gray’s Anatomy: The Anatomical Basis of Clinical Practice. https://shop.elsevier.com/books/grays-anatomy/standring/978-0-443-06684-9.

[4] Tang JB, Lalonde D, Fernandes CH, Sadek AF, Besmens IS (2026). The IFSSH consensus and current guidelines on flexor tendon repairs and reconstruction. J Hand Surg Eur Vol.

[5] Zukawa M, Osada R, Makino H, Kimura T (2019). Wide-awake flexor pollicis longus tendon reconstruction with evaluation of the active voluntary contraction of the ruptured muscle-tendon. Plast Reconstr Surg.

[6] Leibovic SJ (2007). Arthrodesis of the interphalangeal joints with headless compression screws. J Hand Surg Am.

[7] Cox C, Earp BE, Floyd WE 4th, Blazar PE (2014). Arthrodesis of the thumb interphalangeal joint and finger distal interphalangeal joints with a headless compression screw. J Hand Surg Am.

[8] Soong M, van Leerdam R, Guitton TG, Got C, Katarincic J, Ring D (2011). Fracture of the distal radius: risk factors for complications after locked volar plate fixation. J Hand Surg Am.

[9] Kitay A, Swanstrom M, Schreiber JJ, Carlson MG, Nguyen JT, Weiland AJ, Daluiski A (2013). Volar plate position and flexor tendon rupture following distal radius fracture fixation. J Hand Surg Am.

[10] Cook A, Baldwin P, Fowler JR (2020). Incidence of flexor pollicis longus complications following volar locking plate fixation of distal radius fractures. Hand (N Y).

[11] Langdell HC, Emovon EO, Hammert WC (2026). Management options and clinical outcomes after flexor tendon ruptures following volar plating of distal radius fractures. Hand (N Y).

[12] Limthongthang R, Bachoura A, Jacoby SM, Osterman AL (2014). Distal radius volar locking plate design and associated vulnerability of the flexor pollicis longus. J Hand Surg Am.

[13] White BD, Nydick JA, Karsky D, Williams BD, Hess AV, Stone JD (2012). Incidence and clinical outcomes of tendon rupture following distal radius fracture. J Hand Surg Am.

[14] Griffin JW, Chhabra AB (2014). Complications after volar plating of distal radius fractures. J Hand Surg Am.

[15] Cross AW, Schmidt CC (2008). Flexor tendon injuries following locked volar plating of distal radius fractures. J Hand Surg Am.

[16] Tanaka Y, Aoki M, Izumi T, Fujimiya M, Yamashita T, Imai T (2011). Effect of distal radius volar plate position on contact pressure between the flexor pollicis longus tendon and the distal plate edge. J Hand Surg Am.

[17] Zelenski NA, Schmidt E, Chin M, Gittings D, Steinberg D, Hast MW (2021). Flexor pollicis longus tendon wear associated with volar plating: a cadaveric study. J Hand Surg Am.

[18] Agnew SP, Ljungquist KL, Huang JI (2015). Danger zones for flexor tendons in volar plating of distal radius fractures. J Hand Surg Am.

[19] Meyer MA, Benavent KA, Chruscielski CM, Janssen SJ, Blazar PE, Earp BE (2022). The impact of the Soong Index on hardware removal and overall reoperation rates after volar locking plate fixation of distal radius fractures. J Hand Surg Am.

[20] Nunley JA, Rowan PR (1999). Delayed rupture of the flexor pollicis longus tendon after inappropriate placement of the pi plate on the volar surface of the distal radius. J Hand Surg Am.

[21] Klug RA, Press CM, Gonzalez MH (2007). Rupture of the flexor pollicis longus tendon after volar fixed-angle plating of a distal radius fracture: a case report. J Hand Surg Am.

[22] Saitoh S, Hata Y, Murakami N, Nakatsuchi Y, Seki H, Takaoka K (1999). Scaphoid nonunion and flexor pollicis longus tendon rupture. J Hand Surg Am.

[23] Jablon M, Rabin SI (1988). Late flexor pollicis longus tendon rupture due to retained glass fragments. J Hand Surg Am.

[24] Netscher DT, Badal JJ (2014). Closed flexor tendon ruptures. J Hand Surg Am.

[25] Kara A, Celik H, Bankaoglu M, Oc Y, Bulbul M, Sugun TS (2016). Ultrasonic evaluation of the flexor pollicis longus tendon following volar plate fixation for distal radius fractures. J Hand Surg Am.

[26] Posner MA (1983). Flexor superficialis tendon transfers to the thumb--an alternative to the free tendon graft for treatment of chronic injuries within the digital sheath. J Hand Surg Am.

[27] Bachoura A, Prokuski-Lund V, Kane P, Zelouf DS, Osterman AL (2024). Flexor tendon attritional ruptures following distal radius volar plate fixation. J Hand Surg Am.

